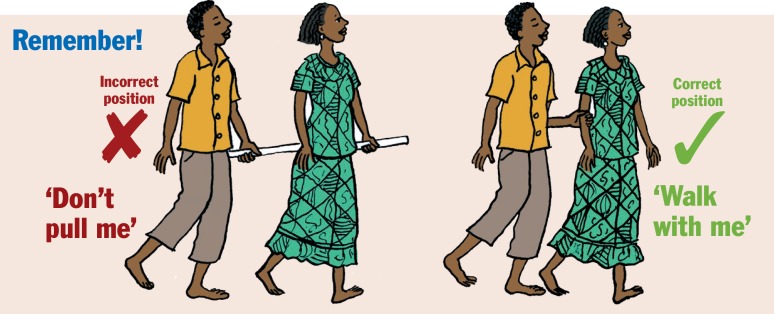# Assisting people who are visually impaired

**Published:** 2016

## Meeting and greeting

Always treat a person with impaired vision as you would anyone else. Introduce yourself first before offering help.

**Figure:**
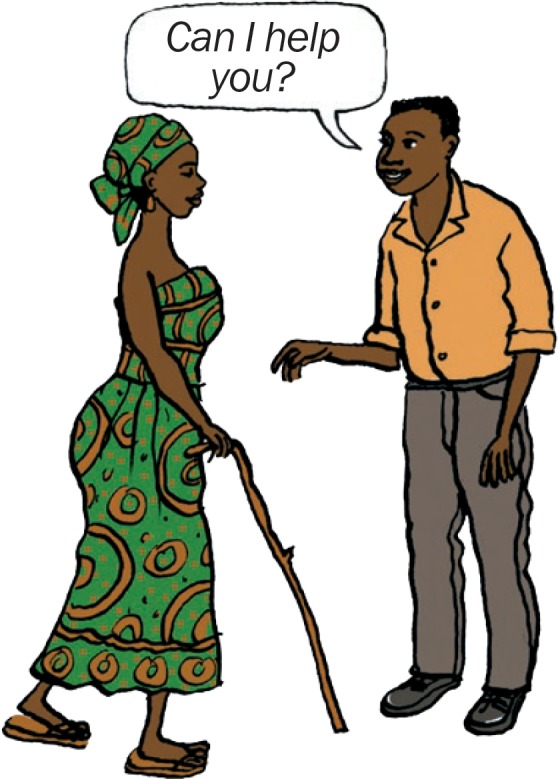


## Guiding

Walk side by side. Allow the person with impaired vision to set the pace and to hold your elbow (hand to elbow).

**Figure:**
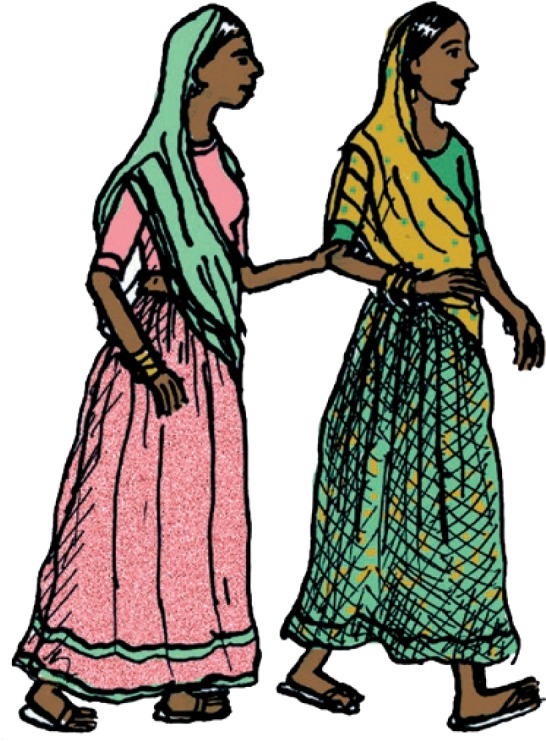


## Steps, stairs and slopes

Tell the blind or visually impaired person whether you are going up or down, and allow time for her or him to hold the handrail. Go one step ahead and take a slightly longer stride on the last step to allow your partner space.

**Figure:**
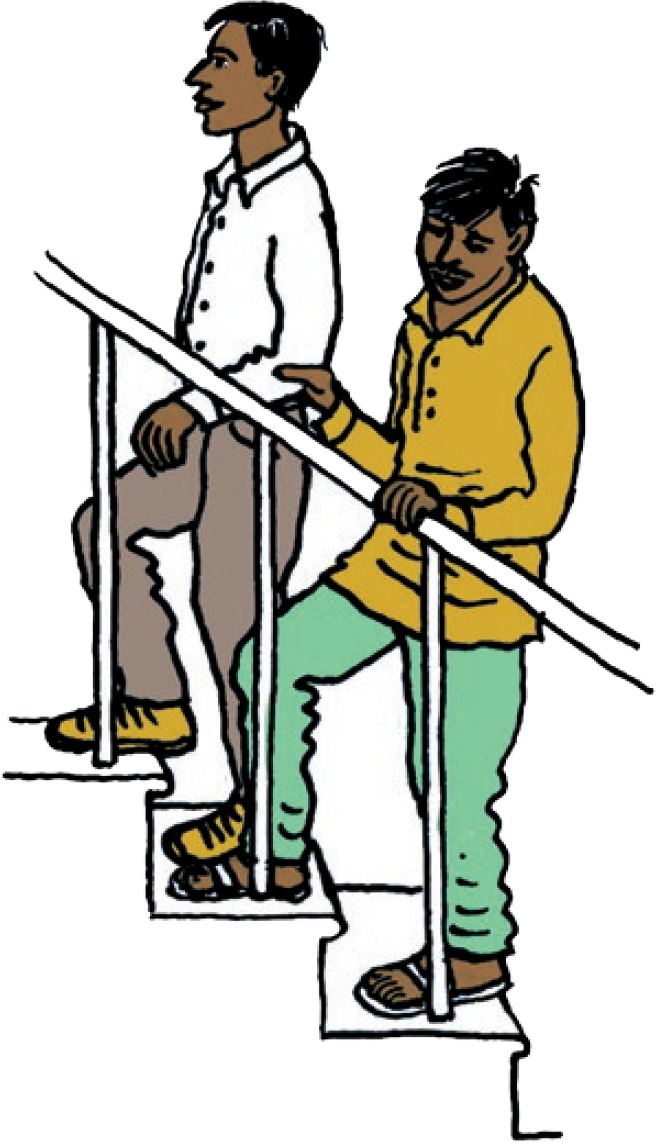


## Doorways

Tell the person whether the door opens towards or away from you. Position yourself so that your partner will pass through the door on the side of the hinge. Open the door with your guiding hand. Allow your partner to feel the handle, follow you through the door, and close the door behind both of you.

**Figure:**
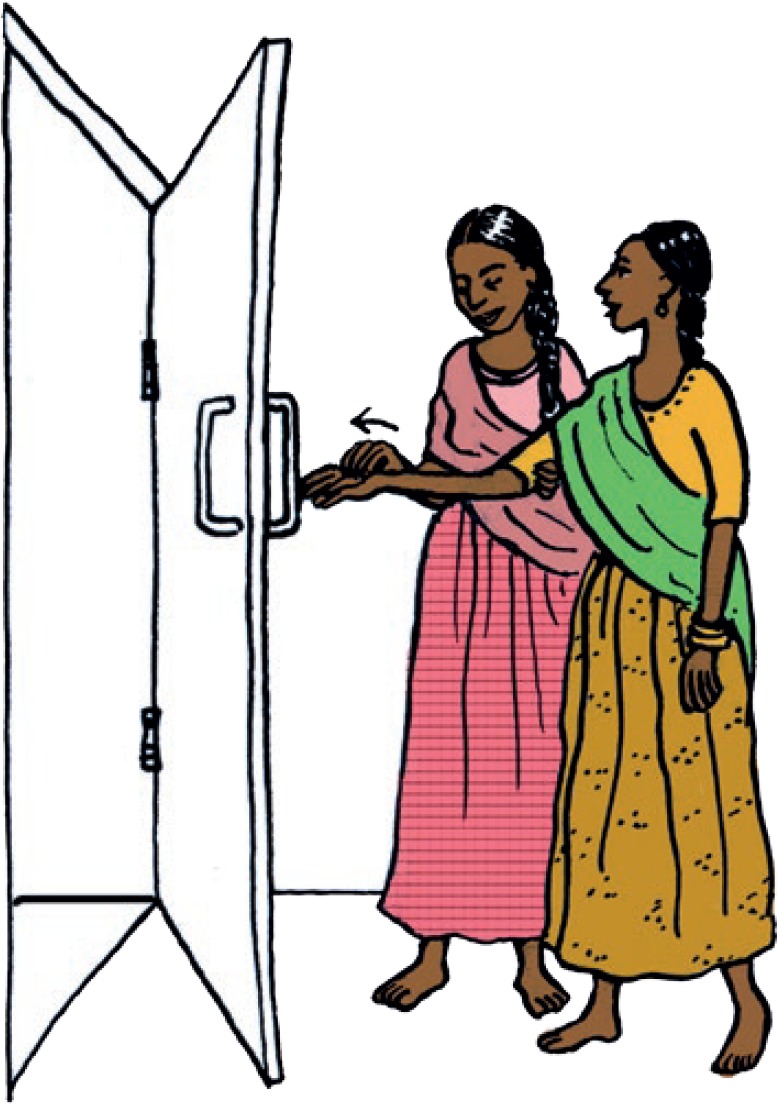


## Seating

Guide the person to the seat and explain what type it is (upright chair, low sofa, armchair, or stool). Ask him to let go of your arm and placea hand on the seat back or on the seat itself. He will now be able to judge its height and sit down safely.

**Figure:**
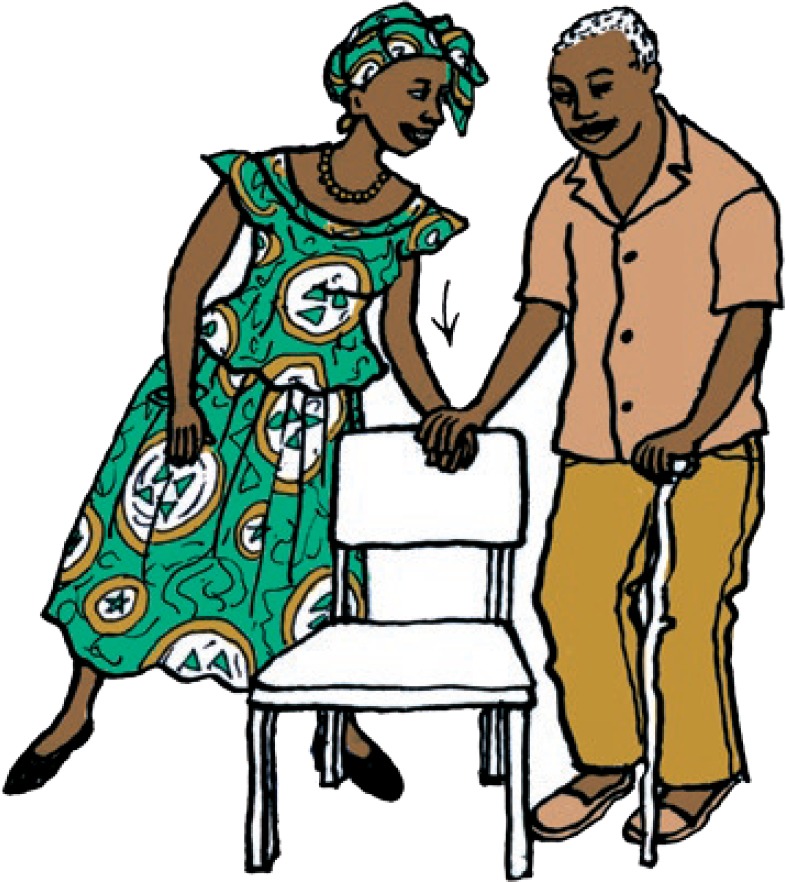


## Narrow spaces

Tell the person about the change in surroundings and then move your own guiding arm towards the middle of your back. Your partner should automatically step in behind you.

**Figure:**
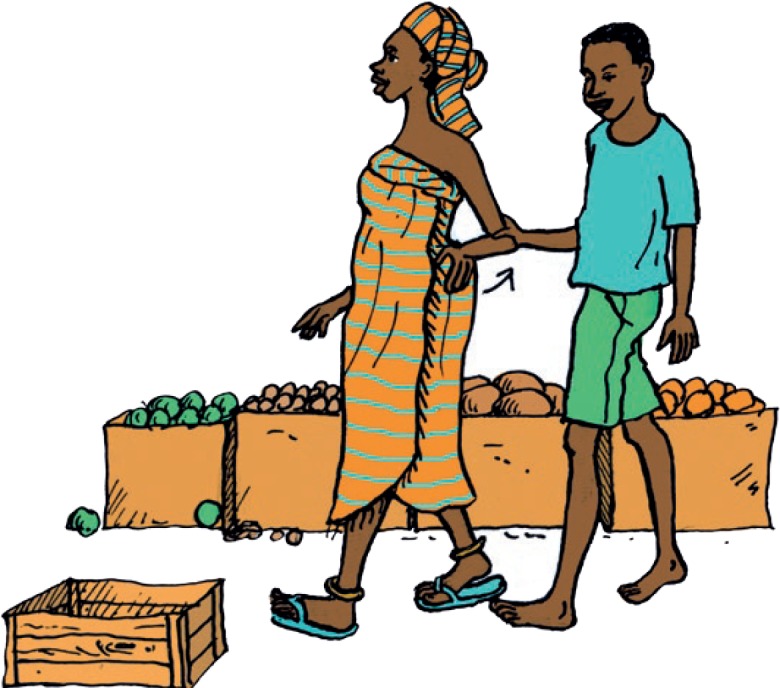


## Roads and kerbs

Tell the person if you are approaching ‘kerb up’ or ‘kerb down’ (the step onto or off a pavement or sidewalk), and pause slightly before taking the step. Cross the road using the shortest distance – usually straight across.

**Figure:**
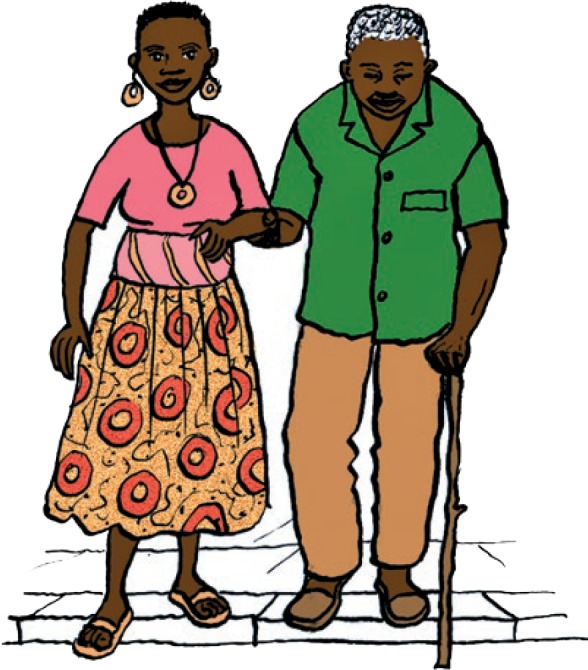


## Travelling by car

**Figure:**
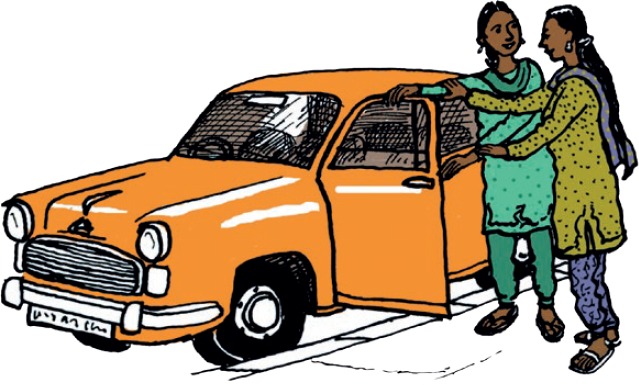


Tell the person if she is getting into the back or the front seat of the car, and whether it is facing left or right. Place your guiding hand on the door handle and allow her to slide her grip hand down your arm to the door handle.

With the other hand she will be able to note the car roof and lower her head appropriately. At the end of the journey, get out first and help your partner out.

**Figure:**